# Accelerated development of rice stripe virus-resistant, near-isogenic rice lines through marker-assisted backcrossing

**DOI:** 10.1371/journal.pone.0225974

**Published:** 2019-12-04

**Authors:** Ju-Won Kang, Dongjin Shin, Jun-Hyeon Cho, Ji-Yoon Lee, Youngho Kwon, Dong-Soo Park, Jong-Min Ko, Jong-Hee Lee

**Affiliations:** Department of Southern Area Crop Science, National Institute of Crop Science, RDA, Miryang, Republic of Korea; Texas Tech University, UNITED STATES

## Abstract

The development of new improved varieties is one of the major goals of plant breeding. Concomitantly, the demand for stable, eco-friendly, and high-quality rice production is constantly increasing. However, most farmers prefer to cultivate familiar rice varieties developed more than 10 years ago to minimize economic risk. A strategy is needed to develop rice varieties without the limitations of the preferred old varieties. Here, we tested the rapid development of near isogenic lines (NILs) using a rapid generation advance system together with marker-assisted backcrossing to overcome the shortcomings of parental materials. For this purpose, we chose rice stripe virus (RSV) susceptible variety Unkwang and RSV resistant variety Haedamssal as experimental materials. First, we backcrossed and screened BC_1_F_1_ and BC_2_F_1_ plants having similar agronomic traits as Unkwang and the heterozygous genotype for RSV resistant specific marker InDel7 from Haedamssal. Secondly, the genetic background of 11 BC_2_F_1_ plants was identified with 73 KASP markers; plants of line YR32548-8 showed 84.5% of recovery of the recurrent parent genome. Among 28 BC_2_F_2_ plants, YR32548-8-16 was the line that showed maximum recovery of the recurrent parent genome (96.2%) while effectively introgressed with RSV-resistance loci on chromosome 11. Finally, we selected line YR32548-8-16 as an NIL showing an RSV resistant phenotype and similar agronomic traits to Unkwang. This fast breeding approach will be useful in rice breeding programs for the improvement of varieties preferred by farmers for their stress tolerance, yield, or quality.

## Introduction

Rice (*Oryza sativa* L.) is the most important staple food crop for more than half of the global population. Diverse rice varieties have been released for cultivation under a range of sunlight, precipitation, and temperature conditions. However, in time, farmers have selected and cultivated only a few of the available rice varieties with the highest yields and best cooking and eating qualities given the cultivation environments of specific growing areas. The choice of rice varieties has greatly influenced their culture, diets, and economic condition. On the other hand, the demand for stable, eco-friendly, and high-quality rice production is increasing. Thus, despite the current availability of various new varieties developed with enhanced disease resistance, abiotic stress tolerance, or high quality characteristics to fulfill both farmer and consumer demands under global climate change and economic and social development, still farmers continue to cultivate only a handful of familiar varieties preferred by consumers, in order to minimize economic risk.

During the last 40 years, molecular marker systems have become well established in rice genetics and breeding. Genome sequence variation analysis using next generation sequencing (NGS) technology that can rapidly generate information regarding high-throughput single nucleotide polymorphism (SNP) occurrence, has been used in attempts to develop effective molecular marker systems. A core set of 768 SNPs was selected for genetic analysis of the *O*. *sativa* ssp. *japonica* rice population [1]. Recently, the Kompetitive allele-specific PCR (KASP) assay was developed as the uniplex SNP-genotyping platform from LGC Genomics (London, UK) [2]. The genotyping panel, consisting of 2,015 KASP assays, was successfully validated for *O*. *glaberrima* and *O*. *sativa* [3]; among them, 1,890 KASP markers were applicable to *indica* rice [4]. Additionally, a core set of 506 KASP markers was constructed for Korean *japonica* rice varieties [5].

Conventional backcross breeding has been used to introduce useful agronomic traits into an elite cultivar or breeding line by repeated crossing to the recurrent parent. Advancement of genomic research in rice has opened new opportunities to reduce the time of backcross breeding. NILs enriched with introduced target traits through backcrossing have been used in practical applications for breeding to improve elite varieties, such as Nipponbare, Minghui63, IR64, Basmati, and Ilmi [6, 7]. Minghui63(*Xa21*) NIL introgressing broad-spectrum bacterial blight resistance gene *Xa21* into Minghui63, was obtained from the BC_3_F_1_ generation after genetic background selection by MAS. Minghui63(*Xa21*) NIL was almost identical with its recurrent parent Minghui63 for agronomic traits such as heading date, plant height and grain characteristics, but not bacterial blight resistance [8]. Saeilmi was developed as an NIL harboring panicle blast resistant gene *Pb1* within the elite Korean panicle blast susceptible variety Ilmi by marker-assisted backcrossing. The agronomic traits of Saeilmi were very similar to those of its parent variety Ilmi, except for panicle blast resistance.

Rice stripe virus (RSV) is one of the most destructive rice viruses, greatly reducing rice production in temperate and subtropical regions of East Asia. RSV is transmitted by the small brown planthopper (*Laodelphax striatellus*, SBPH). Studies have found that RSV does not affect varieties of rice equally, with a larger number of *indica* rice varieties being resistant to RSV than *japonica* varieties [9, 10, 11]. The introduction of RSV resistance genes derived from *indica* varieties has resulted in stable RSV resistance in some *japonica* varieties [11]. Five major RSV resistance QTLs located on chromosome 11 of *O*. *sativa* ssp. *indica* have been reported (*Stv-bi*, *qSTV11*^*IR24*^, *qSTV11*^*TQ*^, *qSTV11*^*KAS*^, and *qSTV11*^*SG*^) [12–16], but have not yet been cloned. *Stv-a* and *Stv-b* have been reported to confer resistance to RSV in Japanese upland rice [17]. *Stv-a* is linked with the waxy gene on chromosome 6, and *Stv-b* is located on chromosome 11. Some *indica* varieties, including Modan, have a different resistance gene, *Stv-bi*, and are incompatible dominance with *Stv-b*. [18–21]. The Kanto PL3 resistance gene derived from the *indica* variety Pe-bi-hun is closely related to the *Stv-b* locus [22]. In the 1960s, *Stv-bi* was transferred from Modan rice to the *japonica* variety Norin 8. The *Stv-bi* gene was subsequently used to breed RSV resistant *japonica* rice varieties in Korea and Japan. In previous studies, we developed an InDel marker that was tightly linked to the *Sty-bi* gene derived from *indica* rice for use in MAS screening [16].

Haedamssal is an RSV resistant (*Stv-bi*) early maturing variety, and Unkwang is widely cultivated as an early maturing variety in Korea, but susceptible to RSV. Herein, we report our demonstration of a rapid and effective backcross breeding strategy using an approach that combined marker-assisted back-crossing (MABC) and rapid generation advance (RGA) to develop RSV-resistant NILs in Unkwang background.

## Materials and methods

### Plant materials

**Fig 1:**
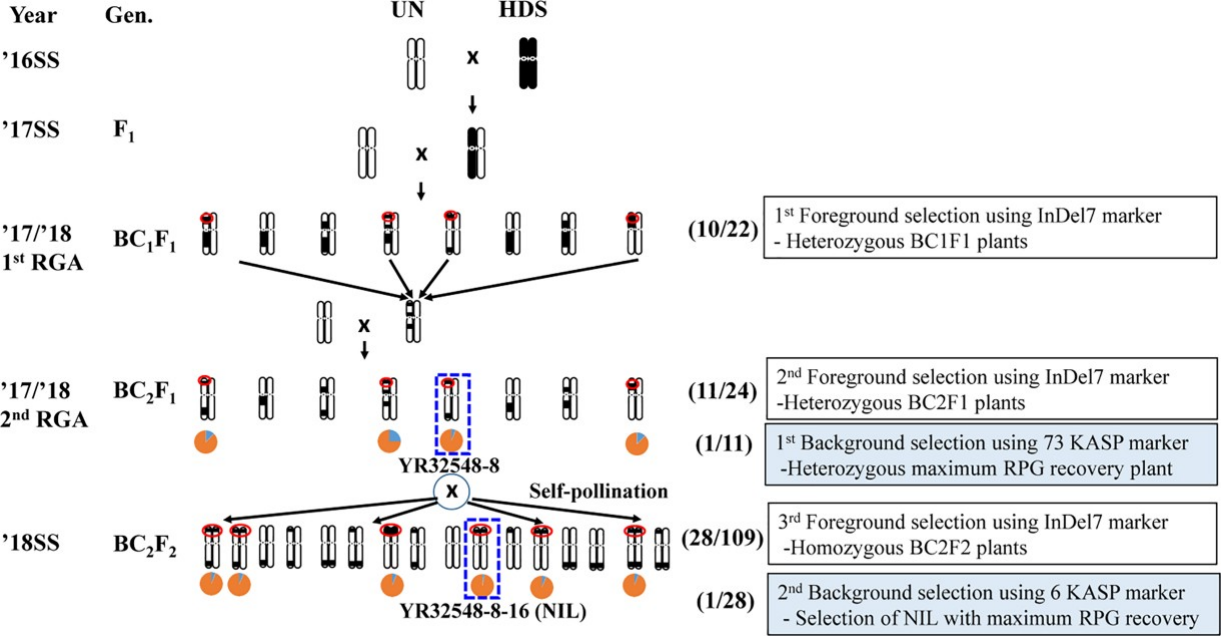
Development of RSV-resistant near isogenic line YR32548-8-16 with details of markers used for foreground and background selection. Numbers in parentheses indicate the total number of plants. SS: summer season, RGA: rapid generation advance in winter season, UN: Unkwang, HDS: Haedamssal.

All plant materials, including the parental varieties, F_1_, and backcross lines, were obtained at the National Institute of Crop Science (NICS), in the City of Miryang, in South Korea. In order to develop a near-isogenic line (NIL) resistant to RSV, Unkwang was used as the recurrent parent for incorporation of RSV resistance genes from Haedamssal, the donor parent, which is a Korean *japonica* rice variety. Unkwang was crossed with Hadamssal to obtain F_1_ seeds (Fig 1). F_1_ plants were backcrossed to Unkwang to obtain BC_1_F_1_ seeds in the summer season and subsequently, heterozygous BC_1_F_1_ plants were transplanted and backcrossed with Unkwang to obtain BC_2_F_1_ seeds in the winter season. The best heterogeneous BC_2_F_1_ plant selected by InDel7 was transplanted to produce the BC_2_F_2_ seeds (Fig 1).

### MABC breeding strategy

For the MABC scheme, Unkwang was crossed with Hadamssal to obtain F_1_ seeds (Fig 1). F_1_ plants were backcrossed to Unkwang to obtain BC_1_F_1_ seeds. Foreground selection for the RSV gene in the BC_1_F_1_ generation was performed using the gene specific molecular marker, InDel7. Twelve heterogeneous individual plants with the desired allele (Aa) were then backcrossed to Unkwang again to produce seeds for the BC_2_F_1_ generation. The second foreground selection cycle was performed in the BC_2_F_1_ generation and then the third foreground selection cycle was conducted in the BC_2_F_2_ generation to select the homozygous allele for the *Stv-bi* locus. On the other hand, background selection in the BC_2_F_1_ and BC_2_F_2_ progenies focused on the best plants to select heterogeneous BC_2_F_1_ plants (Aa) with gene-specific marker InDel7. The first background selection to assess the recovery of the recurrent parent genome (RPG) was performed using 73 polymorphic KASP markers selected from [5], in plants which were to be self-pollinated to produce the seeds of the BC_2_F_2_ generation. The second background selection was conducted to select for maximum recovery of RPG (Fig 1).

### Rapid generation advance under greenhouse conditions

A Rapid Generation Advance (RGA) system allowed us to produce two generations per year in a greenhouse during the winter season in Korea. The first RGA was conducted to grow the BC_1_F_1_ generation from Oct. 1. to Jan. 15, and the second RGA was performed to grow the BC_2_F_1_ generation from Jan 15. to Apr. 20. Plants were grown in a greenhouse at 22°C night temperature under natural short-day conditions in the winter season. BC_2_F_2_ seeds were sown in germination trays in the greenhouse and then seedlings were transplanted to a paddy field for the evaluation of agronomic traits.

### Molecular marker analysis

Total genomic DNA was extracted from fresh leaves of 4-week-old seedlings using the CTAB method with some modifications. DNA was quantified by using nano-drop spectrophotometry (ND1000, Thermo Fisher Scientific, USA). The diluted DNA samples were again diluted with 1X TE buffer (10 mM Tris-HCl, pH 8.0, 1 mM EDTA, pH 8.0) to get a concentration of 5 ng/μL and kept in the refrigerator for PCR analysis. The total PCR reaction volume (15 μL) contained 25 ng of template DNA, 1.0 mM of each primer, 0.8 μl of dNTP, 1.5 μL of 10X buffer, 6.6 μL DDW and 0.1 μL of Taq polymerase. In the case of foreground selection marker, InDel7, PCR amplification was carried out in a thermocycler (Veriti, Applied Biosystems, Paisley, UK) using an initial denaturation at 94°C for 2 min followed by 35 cycles at 95°C for 20 s, 60°C for 40 s, 72°C for 30 s, and a final extension at 72°C for 5 min, followed by rapid cooling to 4°C prior to analysis [16]. For background selection marker, KASP amplifications and allelic discriminations were performed using the Nexar system (LGC Douglas Scientific, Alexandria, USA) in the Seed Industry Promotion Center (Gimje, Korea) of Foundation of Agri. Tech. Commercialization & Transfer in Korea. An aliquot (0.8 μL) of 2 Master Mix (LGC Genomics, London, UK), 0.02 μL of 72 KASP assay mix (LGC Genomics, London, UK), and 5 ng genomic DNA template were mixed in 1.6 μL KASP reaction mixture in a 384-well Array Tape. The reactions were run in duplicate and with non-template controls in each run. KASP amplification was performed using a thermal cycling profile from [5]. Recycling was performed twice, and fluorescence readings were taken for KASP genotyping following PCR [5].

### Evaluation of RSV resistance

Forty seeds were grown in a plastic tray (60 × 30 cm), and 2-week-old seedlings were subsequently placed inside clear plastic cages with the viruliferous insect SBPH, as 2nd or 3rd instar nymphs with a density of approximately seven insects per seedling. After 14 days, the insects were removed, and seedlings were transplanted in a greenhouse in the National Institute of Crop Science (Miryang, Korea). The reaction to RSV was evaluated using the virus resistance bioassay method, by calculating the percentage of healthy plants in a given plot at 1 month after transplanting [11] and the ELISA was conducted using the double antibody sandwich (DAS)-ELISA kit (KisanBio, Seoul, Korea) for RSV with an alkaline phosphatase label [23] by calculating the percentage of negative reaction to RSV.

### Statistical analysis

Chi-squared tests for goodness-of-fit were used to evaluate deviations of the observed data from expected segregation ratios in each backcross generation. In the case of background selection, the marker data was analyzed using Graphical Genotyper 2.0 [24]. The homozygous recurrent allele, the homozygous donor allele, and the heterozygous allele were scored as ''A'', ''B'', and ''H'', respectively.

The recovery of the recurrent parent genome (RPG) was calculated by the percentage of markers homozygous for recipient parent (%A) and half of the plants heterozygous at this locus (%H/2).

The mean difference between agronomic data from the parental lines and RSV resistant improved lines was analyzed by Duncan's multiple range test using the SAS 9.4 program.

## Results

### Rapid and effective selection of introgression lines harboring *Stv-bi* through combined MABC and RGA

**Fig 2:**
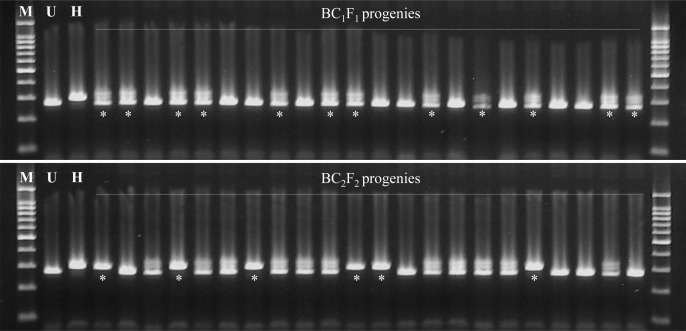
Foreground selection with gene specific marker InDel7 in BC_1_F_1_ and BC_2_F_2_. M: 100 bp ladder, U: recurrent parent Unkwang, H: donor parent Haedamssal, *: selected plants.

To examine a rapid and effective backcross breeding process through marker-assisted foreground selection and background selection, we chose two early maturing Korean rice varieties, Unkwang and Hadamssal, which are RSV-susceptible and RSV-resistant, respectively. Hadamssal is known to possess the *stv-bi* allele on chromosome 11 derived from the Modan variety. First, during the summer season in 2016 we selected five F_1_ plants showing good agronomic phenotype derived from the cross between Unkwang and Hadamssal as recurrent and donor parents, respectively. Then we backcrossed these five F_1_ plants with Unkwang to get BC_1_F_1_ seeds in the summer season in 2017 and grew twenty-two BC_1_F_1_ plants in a greenhouse in the winter season from October 1st 2016 through January 15 2017. When we tested the genotypes of these 22 plants using InDel7, which is tightly linked with RSV resistance for foreground selection, 10 plants showed the heterozygous genotype for InDel7 and 12 plants held the same allele as the susceptible recurrent parent, Unkwang (Fig 2). The segregation ratio was 1:0.83 for the homozygous recurrent allele and the heterozygous allele, which agreed with the expected ratio for the BC_1_F_1_ generation, with a non-significant chi-square value of 0.18 at a probability level of 0.67 (Table 1).

**Table 1:** Foreground selection by gene specific marker InDel7 in BC_1_F_1_, BC_2_F_1_ and BC_2_F_2_ populations.

Crossing No.	Generation	Total number of tested plants	No. of selected plants by InDel7			*X*^*2*^-test		
			A	B	H	Expected value	*X*^*2*^	P-value
YR32533	BC_1_F_l_	22	10	0	12	1 : 1	0.18	0.67
YR32548	BC_2_F_1_	24	13	0	11	1 : 1	0.17	0.68
YR32548-8	BC_2_F_2_	109	28	29	52	1 : 2 : 1	1.98	0.16

A: Unkwang allele, B: Haedamssal allele, H: Heterozygous, R: Recombinant marker showing homozygous donor parent allele.

The five plants in the BC_1_F_1_ generation showing similar agronomic traits as the recurrent parent, such as heading date, plant height, tiller number plus the heterozygous allelic type for InDel7 were selected and backcrossed to Unkwang to obtain BC_2_F_1_ seeds. Twenty-four BC_2_F_1_ plants were grown in a greenhouse from January 15 through April 20 2018. Among these, 11 plants showed the heterozygous allele and 13 plants were identified as RSV-susceptible, as Unkwang. The segregation ratio was 1:0.85 for the homozygous and heterozygous alleles, which again agreed with the expected ratio for the BC_2_F_1_ generation, with a non-significant chi-square value of 0.17 (Table 1). We used 329 KASP markers from the core set of 506 developed for Korean *japonica* rice varieties to examine 11 plants in the BC_2_F_1_ generation for background selection [5]. When we tested 329 KASP markers, 73 KASP markers showed polymorphism between Unkwang and Hadamssal. The number and percentage of selected KASP markers on each chromosome were 1 to 11 and 4.0–52.6%, respectively (S1 Table). We applied these 73 KASP markers to investigate the background of 11 BC_2_F_1_ plants. The residual segments derived from the donor Hadamssal were distributed on all chromosomes, except for chromosomes 3 and 5. Although the number of polymorphic KASP markers on chromosomes 3 and 5 was low, all tested KASP markers showed the same allelic type as the recurrent parent Unkwang. The homozygous substitution segments on 11 of BC_2_F_1_ plants derived from Hadamssal ranged from 0.0–1.1% on the basis of the physical size of the rice chromosomes; concomitantly, the percentage of RPG recovery in the BC_2_F_1_ generation ranged from 75.3 to 84.5%.

**Fig 3:**
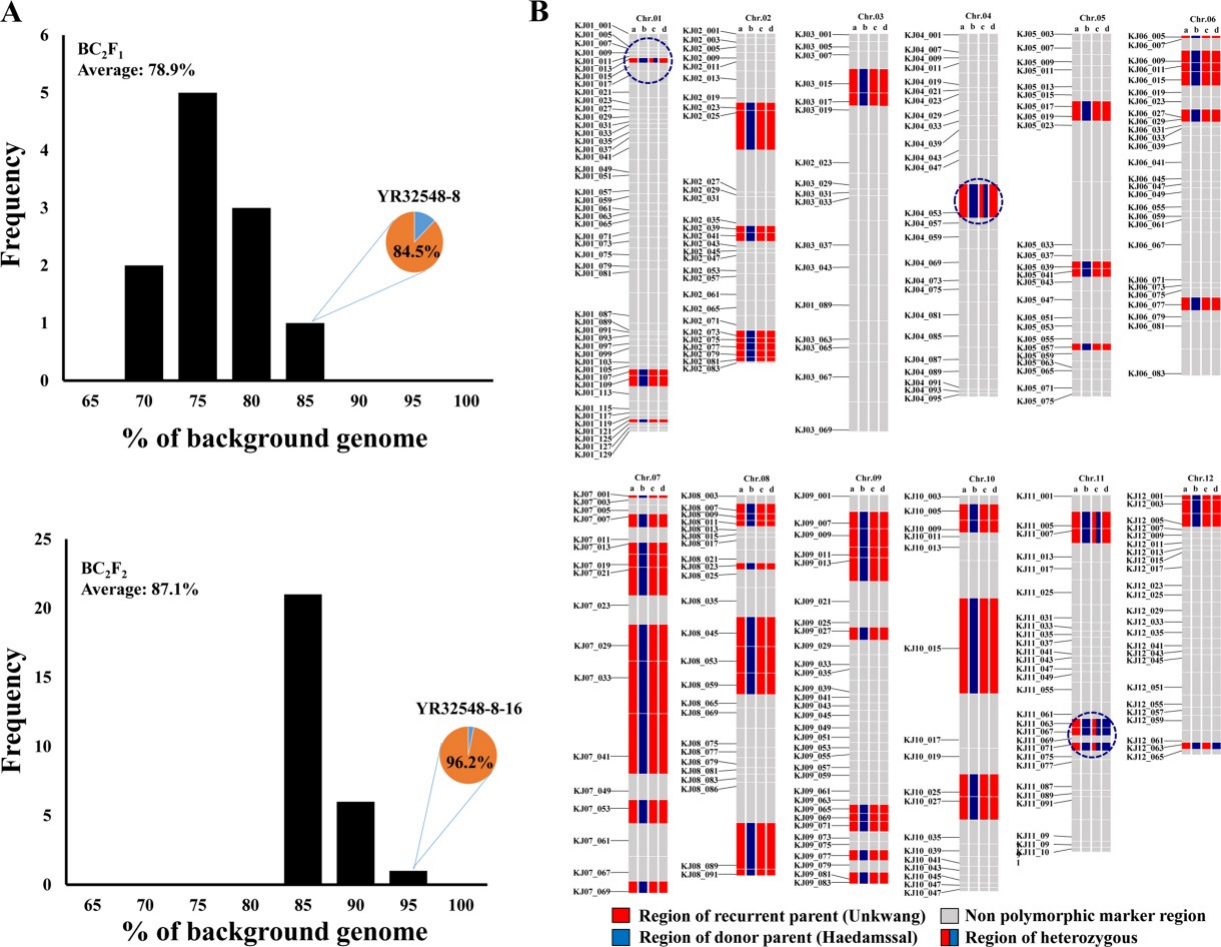
Background selection in BC_2_F_1_ and BC_2_F_2_. A: Frequency of recurrent parent genome recovery in BC_2_F_1_, B: Frequency of recurrent parent recovery in BC_2_F_2_, C: Graphical genotype of selected lines in BC_2_F_1_ and in BC_2_F_2_ and their parents using KASP marker (a: recurrent parent Unkwang, b: donor parent Haedamssal, c: selected BC_2_F_1_ plant YR32548-8, d: the selected BC_2_F_2_ plant YR32548-8-16).

Agronomic characteristics including heading date, plant height, and RPG recovery, are important to develop NILs. Line YR32548-8 showed maximum RPG recovery (84.5%) and no homozygous substitution segments derived from the donor variety, thus containing only heterozygous introgression on chromosomes 1, 4, and 11. Therefore, we chose line YR32548-8 for rapid generation of a NIL (S2 Table) and 109 BC_2_F_2_ seeds were produced by self-pollination of YR32546-8, out of which, 28 plants showed the homozygous resistant haplotype, 29 plants showed the susceptible haplotype, and 52 plants were identified as heterozygous by examination of RSV resistance with the InDel7 molecular marker. The segregation ratio in the BC_2_F_2_ generation was 1:1.86:1.04, with a non-significant chi-square value of 0.17 (Table 1). Next, we carried out a KASP assay targeting for residual segments on chromosomes 1, 4, and 11 to examine the background of the 28 BC_2_F_2_ plants holding the homozygous resistant allele. The percentage of RPG recovery in these plants ranged from 84.0% to 96.2% (Fig 3B, S3 Table). In summary, YR32548-8-16 showed the highest RPG recovery (96.2%) and harbored RSV-resistance loci introgressed only on chromosome 11. Therefore, we designated this YR32548-8-16 as Unkwang-RSV-NIL.

### Validation of agronomic traits and RSV resistance in the selected NIL

**Fig 4:**
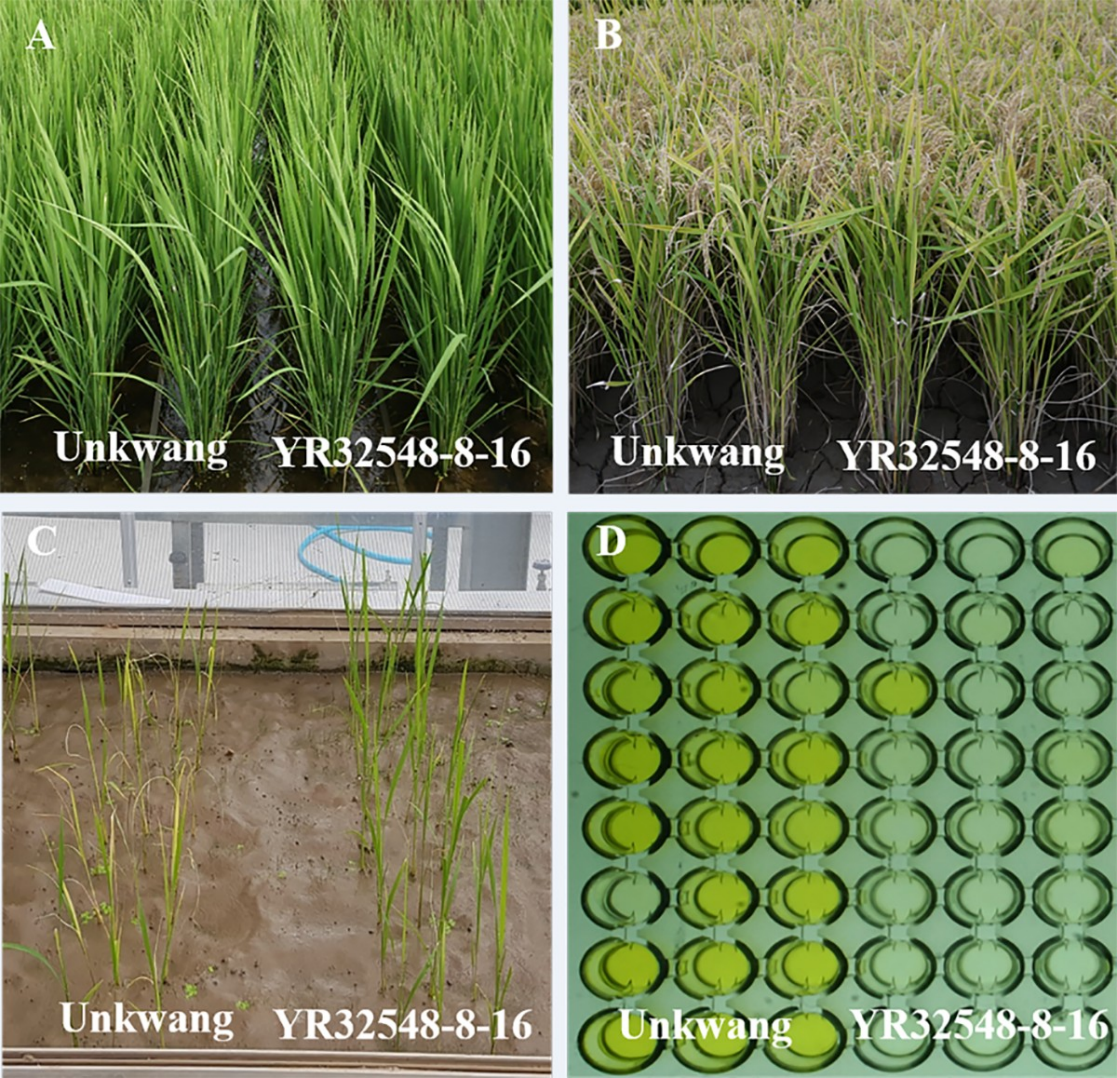
Phenotypic differences between Unkwang and YR32548-8-16. A: Booting stage, B: Ripening stage, C: Reaction to RSV disease, D: ELISA assay.

Most of the agronomic traits of the selected YR32548-8-16 lines, such as days to heading, panicle length, number of spikelets per panicle, and ripening ratio, were similar to the corresponding traits in the recurrent parent (Fig 4A and 4B, S4 Table). The selected NIL has a typical short-grain shape as indicated by the length/width ratio of 2.21 for brown rice (data not shown). RSV resistance conferred by the *Stv-bi* gene in the NIL was introgressed by MABC, as confirmed using the gene-specific InDel7 marker. In an RSV bioassay and ELISA test at the seedling stage, selected NIL YR32548-8-16 showed strong resistance to RSV, whereas Unkwang was highly susceptible to RSV (Fig 4C and 4D).

## Discussion

Rapid and efficient breeding strategies are crucial to developing new rice varieties with good yield and high quality. In this study, we demonstrated a fast breeding technique which shortened the number of cycles of segregating generations through MABC. First, a plant showing similar agronomic attributes as the parent having the target trait after backcross is selected. In a second step, background screening is performed to find a NIL showing maximum RPG recovery possible. Through this simple strategy, we developed one such NIL line in the Unkwang background within three years.

Conventional backcrossing breeding has been used to introduce useful agronomic traits such as disease resistance, but this approach needs at least six cycles of backcross to minimize undesirable linkages in the donor parent [25]. During the last 30 years, diverse molecular markers related to specific traits were reported and used for marker-assisted backcross breeding as foreground selection [26]. Background selection was first applied to introduce the bacterial blight resistant gene *Xa21* in cultivar Minghui 63 [27]. Recently, the foreground and background selection approach for MABC has been widely applied for developing new disease resistant varieties in rice [27–29]. Korean farmers prefer to cultivate Unkwang because of is early maturity and high cooking quality. However, when RSV spread across the Korean peninsula, Unkwang productivity was severely reduced [30]. Therefore, we chose Unkwang to demonstrate fast NIL development by the early advance MABC method.

The population size of each generation is one of main issues for foreground and background selection in MABC, because it is time-consuming and labor intensive. When bacterial blight resistance gene *Xa38* gene was introduced to the improved Samba Mahsuri background, 70 to 160 plants in each generation from F_1_ to BC_3_F_1_ were examined for foreground selection [31]. Around 40 to 45 plants from generations BC_2_F_1_ and BC_3_F_1_ were tested for background selection for NIL-*Xa38* development; 26 and 63 plants of BC_1_F_1_ and BC_2_F_1_ were genotyped with gene-specific pTA248 marker for *Xa21* and genetic background of 8 and 11 plants, respectively, were selected for the development of NIL-*Xa21* in LT2 background [32]. Selection of the donor parent is also critical to minimize the breeding population for MABC. If recurrent and donor parents are very similar genetically and in agronomic traits, except for the target trait, a small population may be sufficient and the period for NIL development might therefore be reduced. However, most researchers use known varieties as donor parents because screening for donor parents possessing the target trait requires a significant amount of time. Therefore, numerous polymorphic markers were identified and used for background selection. Eighty-three SSR markers among the 366 on chromosome 12 that were polymorphic between ISM and donor parent PR114(*Xa38*) were used to introduce the *Xa38* gene [31]. Similarly, 119 and 95 of polymorphic markers on chromosomes 12 and 4, respectively, were used to develop NIL-*Bph9* and LuoYang69, which were created by pyramiding of *Bph6* and *Bph9*, respectively [33]. In a previous study, we narrowed down the loci for RSV resistance and developed molecular marker InDel7 which is tightly linked to RSV resistance [16, 34]. This allowed us to reduce the population size for MAS in foreground selection without phenotypic selection on target traits. Thus, we screened RSV-resistant variety Hadamssal with early maturity [35]. Most agronomic traits including heading date and plant height are highly similar between Unkwang and Hadamssal, except for RSV resistance. Therefore, we chose Hadamssal as the RSV resistant donor and identified 12 and 11 plants in BC_1_F_1_ and BC_2_F_1_, respectively, showing the heterozygous genotype using the InDel7 marker.

Background selection for rapid RPG recovery will be extremely useful if backcrossing is continued in a greenhouse or under a non-target environment [36]. Until recently, conventional gel-based markers including SSR and SNP markers were widely used for background selection for RPG recovery in MABC [37–39]. These marker systems showed low resolution and density for whole genome coverage and were labor-intensive and time-consuming. Whole genome sequencing approaches are useful to investigate the genetic background in MABC. Despite the availability of several technologies, such as genotyping by sequencing (GBS), whose improvement has reduced the experimental cost of whole genome sequencing of plant materials, the technique is still high-cost and time-consuming for useful application in rice breeding fields [40]. On the other hand, the KASP marker system that uses fluorescence for genotyping has proved to be a cost-effective, single-step genotyping technology that is more flexible than GBS or chip-based genotyping [4, 41]. Recently, 506 KASP markers based on SNP were established for genotyping Korean *japonica* rice varieties by another research group working at our institute [5]. We applied this 506 KASP-marker-set for rapid background selection at each generation and identified 22% of KASP markers showing polymorphism between Unkwang and Hadamssal. Most marker-assisted background selection protocols are commonly conducted in segregating generations, such as BC_1_F_1_, BC_2_F_1_, and BC_3_F_1_, and the selected lines are then advanced up to a BC_n_F_5_ generation for genetic fixation [39]. In this study, the percentage of RPG recovery in the BC_2_F_1_ generation ranged from 74.6% to 85.6%, with line YR32548-8 showing maximum RPG recovery. Among 28 plants screened for the homozygous resistant genotype in the BC_2_F_2_ generation, line YR32548-8-16, showing 96.2% recovery of RPG, was screened through second background selection (S3 Table). This high RPG recovery rate was approximately 9% higher than the theoretical RPG recovery rate for BC_2_F_2_, the reason being that we had performed background selection twice already. Specifically, target loci for RSV on chromosome 11 were introduced in line YR32548-8-16.

Rapid generation advance techniques are an imperative requirement to shorten the time needed to develop breeding lines. In temperate countries where rice can only be grown during one season each year due to extreme winter cold, RGA would significantly reduce the time needed for breeding by off-season generation advancement. In Korea, RGA has been used for high-quality *japonica* rice breeding since the 1960s [42]. In this study, we cultivated two generations (BC_1_F_1_ and BC_2_F_1_) per year in a greenhouse. The conventional breeding method usually takes 3–4 years to develop genetically fixed lines [43] but, ultimately, we developed homozygous NILs in the BC_2_F_2_ generation within three years and our selected NILs showed a high level of resistance against RSV, with an infection ratio lower than 30% (Fig 4D). Initially, the cost of MABC would be higher, compared to the conventional breeding in the short-term, but this would be balanced by the amount of time saved. This is a major concern for rice breeders, as the accelerated release of an improved variety may translate into earlier farmer profit.

## Conclusions

We successfully developed an RSV-NIL of Unkwang using a combined MABC-RGA approach to incorporate RSV resistance under control by one major gene. The RPG recovery was greatly accelerated by foreground selection using gene specific marker InDel7, linked to the target resistance, and background selection of high throughput KASP markers derived from SNP of Korean japonica rice varieties. The use of RGA shortened the period for developing NILs by at least three years, compared to conventional backcross breeding. More importantly, considering the agricultural characteristics of donor parents, the size of the donor chromosome segment containing the target locus has been reduced to ensure minimal changes in the genetic makeup of the recurrent parent.

## Supporting information

S1 Table Percentage of polymorphic markers used in this study. (DOCX)

S2 Table Analysis of the first background selection in BC_2_F_1_ by KASP markers. (DOCX)

S3 Table Analysis of the second background selection and introgression segment from donor parent in the selected BC_2_F_2_ population by KASP markers. (DOCX)

S4 Table Comparison of agronomic traits between YR32548-8-16 and parent. (DOCX)
