# Supplementary material for: Accelerated development of rice stripe virus-resistant, near-isogenic rice lines through marker-assisted backcrossing
Source: PLoS One. 2019 Dec 4;14(12):e0225974. doi: 10.1371/journal.pone.0225974 (PMC6892552; doi:10.1371/journal.pone.0225974)
Supplement: S1 Table — (DOCX) [file pone.0225974.s001.docx]

**Supporting information**

**S1 Table. Percentage of polymorphic markers used in this study.**

| Chromosome | No. of KASP markers | | | |
| --- | --- | --- | --- | --- |
|  | Total | Polymorphic | non-polymorphic | Percentage of polymorphism (%) |
| 1 | 62 | 4 | 58 | 6.5 |
| 2 | 29 | 9 | 20 | 31.0 |
| 3 | 17 | 2 | 15 | 11.8 |
| 4 | 25 | 1 | 24 | 4.0 |
| 5 | 24 | 5 | 19 | 20.8 |
| 6 | 28 | 7 | 21 | 25.0 |
| 7 | 19 | 10 | 9 | 52.6 |
| 8 | 24 | 11 | 13 | 45.8 |
| 9 | 32 | 11 | 21 | 34.4 |
| 10 | 17 | 5 | 12 | 29.4 |
| 11 | 28 | 4 | 24 | 14.3 |
| 12 | 24 | 4 | 20 | 16.7 |
| Total | 329 | 73 | 256 | 22.2 |
