# Supplementary material for: Accelerated development of rice stripe virus-resistant, near-isogenic rice lines through marker-assisted backcrossing
Source: PLoS One. 2019 Dec 4;14(12):e0225974. doi: 10.1371/journal.pone.0225974 (PMC6892552; doi:10.1371/journal.pone.0225974)
Supplement: S2 Table — (DOCX) [file pone.0225974.s002.docx]

**S3 Table. Analysis of the first background selection in BC_2_F_1_ by KASP markers.**

| Plant No. | Genetic background (%) | | |  | No. of introgression marker | |  | | Chromosome of introgression |
| --- | --- | --- | --- | --- | --- | --- | --- | --- | --- |
|  | A | B | H |  | R | H |  |  |  |
| YR32548-1 | 82.9 | 0.0 | 17.1 |  | 0 | 9 |  | | 1, 2, 6, 11 |
| YR32548-2 | 81.3 | 0.4 | 18.3 |  | 1 | 10 |  | | 1, 2, 6, 7, 9, 11, 12 |
| YR32548-3 | 75.3 | 1.1 | 23.6 |  | 2 | 20 |  | | 2, 4, 6, 7, 9, 10, 11, 12 |
| YR32548-4 | 78.2 | 0.0 | 21.8 |  | 0 | 15 |  | | 1, 2, 6, 7, 10, 11, 12 |
| YR32548-5 | 76.5 | 0.0 | 23.5 |  | 0 | 16 |  | | 2, 6, 7, 10, 11, 12 |
| YR32548-6 | 75.3 | 0.4 | 24.3 |  | 1 | 19 |  | | 1, 2, 6, 7, 9, 11, 12 |
| YR32548-7 | 82.7 | 0.4 | 16.9 |  | 1 | 16 |  | | 2, 4, 6, 9, 10, 11 |
| YR32548-8 | 84.5 | 0.0 | 15.5 |  | 0 | 6 |  | | 1, 4, 11 |
| YR32548-9 | 77.3 | 1.1 | 21.6 |  | 2 | 17 |  | | 2, 4, 6, 7, 10, 11 |
| YR32548-10 | 78.6 | 0.4 | 21.0 |  | 1 | 11 |  | | 1, 6, 9, 10, 11, 12 |
| YR32548-11 | 75.3 | 0.0 | 24.7 |  | 0 | 16 |  | | 1, 2, 6, 7, 9, 11, 12 |
| Average | 78.9 | 0.4 | 20.8 |  | 0.74 | 14.1 |  | |  |

A: Unkwang allele, B: Haedamssal allele, H: Heterozygous, R: Recombinant marker showing homozygous donor parent allele
