# Supplementary material for: Accelerated development of rice stripe virus-resistant, near-isogenic rice lines through marker-assisted backcrossing
Source: PLoS One. 2019 Dec 4;14(12):e0225974. doi: 10.1371/journal.pone.0225974 (PMC6892552; doi:10.1371/journal.pone.0225974)
Supplement: S3 Table — (DOCX) [file pone.0225974.s003.docx]

**S4 Table. Analysis of the second background selection and introgression segment from donor parent in the selected BC_2_F_2_ population by KASP markers**.

| Plant No. | Genetic background (%) | | |  | No. of introgression marker | |  | Chromosome of introgression | |
| --- | --- | --- | --- | --- | --- | --- | --- | --- | --- |
|  | A | B | H |  | R | H |  |  |  |
| YR32548-8-1 | 84.6 | 7.3 | 8.1 |  | 4 | 2 |  | 1, 4, 11 |  |
| YR32548-8-2 | 84.6 | 14.0 | 1.4 |  | 4 | 2 |  | 1, 4, 11 |  |
| YR32548-8-3 | 84.6 | 11.9 | 3.4 |  | 4 | 2 |  | 1, 4, 11 |  |
| YR32548-8-4 | 88.1 | 5.1 | 6.8 |  | 3 | 1 |  | 1, 4, 11 |  |
| YR32548-8-5 | 91.4 | 3.8 | 4.8 |  | 2 | 3 |  | 4, 11 |  |
| YR32548-8-6 | 91.4 | 3.8 | 4.8 |  | 2 | 3 |  | 4, 11 |  |
| YR32548-8-7 | 84.6 | 5.1 | 10.2 |  | 3 | 3 |  | 1, 4, 11 |  |
| YR32548-8-8 | 84.6 | 3.8 | 11.6 |  | 2 | 4 |  | 1, 4, 11 |  |
| YR32548-8-9 | 84.6 | 11.9 | 3.4 |  | 4 | 2 |  | 1, 4, 11 |  |
| YR32548-8-10 | 88.1 | 3.8 | 8.1 |  | 2 | 2 |  | 1, 4, 11 |  |
| YR32548-8-11 | 84.6 | 3.8 | 11.6 |  | 2 | 4 |  | 1, 4, 11 |  |
| YR32548-8-12 | 84.6 | 8.6 | 6.8 |  | 5 | 1 |  | 1, 4, 11 |  |
| YR32548-8-13 | 85.9 | 7.3 | 6.8 |  | 4 | 1 |  | 1, 11 |  |
| YR32548-8-14 | 84.6 | 5.1 | 10.2 |  | 3 | 3 |  | 1, 4, 11 |  |
| YR32548-8-15 | 84.6 | 4.8 | 10.6 |  | 2 | 4 |  | 1, 4, 11 |  |
| YR32548-8-16 | 96.2 | 3.8 | 0.0 |  | 2 | 0 |  | 11 |  |
| YR32548-8-17 | 91.4 | 5.1 | 3.4 |  | 3 | 2 |  | 4, 11 |  |
| YR32548-8-18 | 84.7 | 5.1 | 10.2 |  | 3 | 2 |  | 1, 4, 11 |  |
| YR32548-8-19 | 88.1 | 10.6 | 1.3 |  | 3 | 1 |  | 1, 4, 11 |  |
| YR32548-8-20 | 91.4 | 3.5 | 5.1 |  | 1 | 4 |  | 4, 11 |  |
| YR32548-8-21 | 88.1 | 3.8 | 8.1 |  | 2 | 2 |  | 1, 4, 11 |  |
| YR32548-8-22 | 84.6 | 7.3 | 8.1 |  | 4 | 2 |  | 1, 4, 11 |  |
| YR32548-8-23 | 84.6 | 3.8 | 11.6 |  | 2 | 4 |  | 1, 4, 11 |  |
| YR32548-8-24 | 92.7 | 3.8 | 3.4 |  | 2 | 2 |  | 11 |  |
| YR32548-8-25 | 84.6 | 5.1 | 10.2 |  | 3 | 3 |  | 1, 4, 11 |  |
| YR32548-8-26 | 84.6 | 14.1 | 1.3 |  | 5 | 1 |  | 1, 4, 11 |  |
| YR32548-8-27 | 91.4 | 7.3 | 1.3 |  | 4 | 1 |  | 4, 11 |  |
| YR32548-8-28 | 84.6 | 11.9 | 3.4 |  | 4 | 2 |  | 1, 4, 11 |  |
| Average | 87.1 | 6.6 | 6.3 |  | 3.0 | 2.3 |  |  |  |

A: Unkwang allele, B: Haedamssal allele, H: Heterozygous, R: Recombinant marker showing homozygous don or parent allele.
