# Supplementary material for: Accelerated development of rice stripe virus-resistant, near-isogenic rice lines through marker-assisted backcrossing
Source: PLoS One. 2019 Dec 4;14(12):e0225974. doi: 10.1371/journal.pone.0225974 (PMC6892552; doi:10.1371/journal.pone.0225974)
Supplement: S4 Table — (DOCX) [file pone.0225974.s004.docx]

**S2 Table. Comparison of agronomic traits between YR32548-8-16 and parent.**

| Traits | Haedamssal | Unkwang | YR32548-8-16 |
| --- | --- | --- | --- |
| Days to heading | Aug.18^a*^ | Aug.18^a^ | Aug.18^a^ |
| Culm length (cm) | 65.4^a^ | 65.5^a^ | 63.9^a^ |
| Panicle length (cm) | 21.9^a^ | 21.8^a^ | 21.7^a^ |
| Panicle number (no.) | 14.7^a^ | 14.5^a^ | 13.3^a^ |
| Spikelet per panicle (no.) | 103.2^b^ | 129.3^a^ | 128.1^a^ |
| Fertility ratio (%) | 75.2^a^ | 75^a^ | 74.8^a^ |
| Grain length (mm) | 5.41^a^ | 5.22^b^ | 5.24^b^ |
| Grain width (mm) | 2.71^b^ | 2.79^a^ | 2.81^a^ |
| Grain thickness (mm) | 1.98^b^ | 2.09^a^ | 2.1^a^ |
| 1000-grain weight (g) | 23.6^b^ | 23.8^b^ | 24.4^a^ |

*: Duncan’s multiple range test
